# High-resolution structure of a retroviral protease folded as a monomer

**DOI:** 10.1107/S0907444911035943

**Published:** 2011-10-19

**Authors:** Miroslaw Gilski, Maciej Kazmierczyk, Szymon Krzywda, Helena Zábranská, Seth Cooper, Zoran Popović, Firas Khatib, Frank DiMaio, James Thompson, David Baker, Iva Pichová, Mariusz Jaskolski

**Affiliations:** a Department of Crystallography, Faculty of Chemistry, A. Mickiewicz University, 60-780 Poznan, Poland; b Center for Biocrystallographic Research, Institute of Bioorganic Chemistry, Polish Academy of Sciences, 61-704 Poznan, Poland; c Institute of Organic Chemistry and Biochemistry, Academy of Sciences of the Czech Republic, 166 10 Prague, Czech Republic; d Department of Computer Science and Engineering, University of Washington, Box 352350, Seattle, WA 98195, USA; e Department of Biochemistry, University of Washington, Box 357350, Seattle, WA 98195, USA

**Keywords:** Mason–Pfizer monkey virus, HIV-1, retroviral proteases, retropepsins, protein folding, dimerization inhibition, drug-design targets, AIDS

## Abstract

Mason–Pfizer monkey virus (M-PMV), a D-type retrovirus assembling in the cytoplasm, causes simian acquired immuno­deficiency syndrome (SAIDS) in rhesus monkeys. Its pepsin-like aspartic protease (retropepsin) is an integral part of the expressed retroviral polyproteins. As in all retroviral life cycles, release and dimerization of the protease (PR) is strictly required for polyprotein processing and virion maturation. Biophysical and NMR studies have indicated that in the absence of substrates or inhibitors M-PMV PR should fold into a stable monomer, but the crystal structure of this protein could not be solved by molecular replacement despite countless attempts. Ultimately, a solution was obtained in *mr-rosetta* using a model constructed by players of the online protein-folding game *Foldit*. The structure indeed shows a monomeric protein, with the N- and C-termini completely disordered. On the other hand, the flap loop, which normally gates access to the active site of homodimeric retropepsins, is clearly traceable in the electron density. The flap has an unusual curled shape and a different orientation from both the open and closed states known from dimeric retropepsins. The overall fold of the protein follows the retropepsin canon, but the C^α^ deviations are large and the active-site ‘DTG’ loop (here NTG) deviates up to 2.7 Å from the standard con­formation. This structure of a monomeric retropepsin determined at high resolution (1.6 Å) provides important extra information for the design of dimerization inhibitors that might be developed as drugs for the treatment of retroviral infections, including AIDS.

## Abstract 2

The crystal structure of Mason–Pfizer monkey virus protease folded as a monomer has been solved by molecular replacement using a model generated by players of the online game *Foldit*. The structure shows at high resolution the details of a retroviral protease folded as a monomer which can guide rational design of protease dimerization inhibitors as retroviral drugs.

## 1. Introduction

Mason–Pfizer monkey virus (M-PMV), or simian retrovirus type 3 (SRV-3), is a D-type retrovirus (assembling in the cytoplasm of the infected cell) that causes simian acquired immunodeficiency syndrome (SAIDS) in Asian monkeys of the genus *Macaca*. Its protease (PR), which is necessary for the processing of, but is also an integral part of, the expressed retroviral fusion polyproteins, is autocatalytically excised as a 17 kDa form that undergoes further C-terminal processing to a 13 kDa (13PR) form. Protease activation and Gag processing must be highly regulated in M-PMV, since the PR remains inactive as part of the Gag-Pro and Gag-Pro-Pol polyproteins until a late stage of virus release from the cell. The C-terminal part, which contains a glycine-rich region called the G-patch (Bauerová-Zábranská *et al.*, 2005 ▶; Svec *et al.*, 2004 ▶), is not necessary for PR activity but is indispensable for the activity of reverse transcriptase (RT) and for virus infectivity, and most probably functions as the N-terminus of RT after the proteolytic cleavage of the Gag-Pro-Pol polyprotein (Krizova *et al.*, unpublished results). *In vitro*, C-terminal autoprocessing of 13PR proceeds even further, yielding a 12 kDa (12PR) form of reduced activity (Zábranský *et al.*, 1998 ▶). M-PMV (and also HIV) PR is activated under reducing conditions in a process that is likely to involve Cys residues in the retroviral Gag polyprotein. In its active form, retroviral PR is a pepsin-like homodimeric enzyme (retropepsin) with an active site com­posed of two DTG loops, each contributing one aspartate to a water-molecule-bound nucleophilic element (Wlodawer *et al.*, 1989 ▶). The integrity of a retropepsin homodimer is maintained by a β-sheet interface woven from alternating N-termini and C-­termini of the subunits, with additional contacts contributed by two flexible flap loops and the catalytic triads themselves.

Since the elucidation of its structure, HIV-1 PR has become the most studied target for rational drug design; indeed, there are now ten PR inhibitors that are used in the clinical treatment of AIDS, which act as substrate analogues blocking the active site of the enzyme. However, the emergence of drug-resistant mutants calls for alternative strategies; the disruption of PR dimerization would be an attractive possibility (Koh *et al.*, 2007 ▶) as it would not interfere with the functioning of host aspartic proteases, which are single-chain proteins. However, this potential drug-design approach has so far been unsuccessful. Therefore, systems such as M-PMV PR, in which the regulation of PR activity is important for virus replication and has been better studied, might benefit efforts aimed at inhibiting HIV-1 PR dimerization and the development of a new generation of drugs for the treatment of AIDS. Indeed, bio­physical experiments have indicated that M-PMV 13PR should form a monomer-dominated equilibrium (shifted towards the dimer in the presence of substrate/inhibitor), in agreement with the NMR structure of the 12PR variant (Veverka *et al.*, 2003 ▶).

In the present study, we used a 13PR protein (Trp1–Ala114) with C7A/C106A/D26N mutations. The Cys→Ala substitutions remove the possibility of uncontrolled S–S aggregation and mimic the Cys-activated PR *in vivo*. The D26N substitution changes the PR active site DTG triplet to prevent autodigestion. The protein could be crystallized in several crystal forms. Some of the crystals were obtained in the presence of an inhibitor added as a dimerization ‘bait’ with the intention of making the crystal structure amenable to molecular-replacement (MR) methods. The best crystals (monoclinic *P*2_1_), used in this study, with an estimated two protein molecules in the asymmetric unit, were grown in the presence of a 1.2-fold molar excess of a peptidomimetic inhibitor. However, the crystal structure resisted all MR attempts, which utilized all available programs and existing crystallographic models of retropepsins (full dimers and individual subunits). The NMR model of monomeric 12PR could also not be used to solve the crystal structure. The *mr-rosetta* algorithm, which has an outstanding record of success with difficult structures, also failed to produce a solution using the existing models (DiMaio *et al.*, 2011 ▶). This daunting protein-folding problem was therefore presented as a challenge to *Foldit* (Cooper *et al.*, 2010 ▶) players, who generated over one million models starting from the NMR coordinates. One of these solutions, when submitted to MR calculations in *mr-rosetta* (DiMaio *et al.*, 2011 ▶), did produce a plausible crystal structure (Khatib *et al.*, 2011 ▶) that could be easily refined to an *R* factor of 0.169 with excellent geometry. The details of the success of the *Foldit*–*Rosetta* approach using a computer-game-derived model have been described elsewhere (Khatib *et al.*, 2011 ▶).

## 2. Materials and methods

### 2.1. Cloning, expression and purification of C7A/C106A/D26N 13PR

The mutations were introduced into the previously described plasmid pBPS13ATG using the QuikChange Site-Directed Mutagenesis Kit (Stratagene; Zábranská *et al.*, 2007 ▶) and verified by DNA sequencing. The expression of M-PMV PR was carried out in *Escherichia coli* BL21 (DE3) cells under previously described conditions (Zábranská *et al.*, 2007 ▶). The protease, which was expressed in inclusion bodies, was renatured by solubilization in 8 *M* urea and stepwise dialysis against 50 m*M* Tris–HCl pH 7.0, 1 m*M* EDTA, 0.05% β-mercaptoethanol (buffer *A*) and was purified by ion-exchange chromatography (batch method) on QAE-Sephadex A-25 equilibrated with buffer *A*.

### 2.2. Crystallization

Prior to crystallization experiments, the protein was incubated overnight with a 1.2-fold molar excess (relative to dimeric enzyme) of a peptidomimetic inhibitor with the sequence Pro-Tyr-Val-Pst-Ala-Met-Thr, where Pst is (3*S*,4*S*)-4-amino-3-hydroxy-5-phenylpentanoic acid, and a *K*_i_ of 5.3 n*M* for wt 13PR protein. Crystallization screens were set up manually using Crystal Screen and Crystal Screen 2 (Hampton Research; Jancarik & Kim, 1991 ▶) and the hanging-drop vapour-diffusion technique at 292 K by mixing 1 µl protein solution (8.5 mg ml^−1^ in 10 m*M* Tris pH 8.5) and 1 µl reservoir solution. Crystals grew to dimensions of 0.3 × 0.15 × 0.15 mm within two weeks over a reservoir solution consisting of 0.1 *M* imidazole pH 6.5 and 1 *M* sodium acetate. For cryoprotection, the crystal was transferred to a solution consisting of the crystallization mother liquor supplemented with 15%(*v*/*v*) glycerol.

### 2.3. Data collection and processing

X-ray diffraction data were collected at 100 K on a MAR CCD 165 mm detector system using synchrotron radiation on EMBL/DESY (Hamburg) beamline X13. Integration, scaling and merging of the intensity data was carried out in the *XDS* package (Kabsch, 2010 ▶). The unit-cell parameters and Bravais lattice were determined using the *COLSPOT* and *IDXREF* subroutines in *XDS*. The intensities were reduced to structure-factor amplitudes by the method of French & Wilson (1978 ▶) and then converted to MTZ format using the *F*2*MTZ* and *CAD* routines of *CCP*4 (Winn *et al.*, 2011 ▶). Space group, unit-cell and data-collection parameters are summarized in Table 1 ▶.

### 2.4. Structure solution and refinement

A model generated by *Foldit* (Cooper *et al.*, 2010 ▶) players (Khatib *et al.*, 2011 ▶) from the NMR coordinates 1nso (Veverka *et al.*, 2003 ▶) successfully solved the structure in *mr-rosetta* (DiMaio *et al.*, 2011 ▶). An initial atomic model of the structure was autobuilt and refined in *PHENIX* (Adams *et al.*, 2010 ▶). Manual rebuilding of the model and divining of water molecules was performed in *Coot* (Emsley & Cowtan, 2004 ▶). Maximum-likelihood structure refinement was carried out in *PHENIX* (Adams *et al.*, 2010 ▶) using all intensity data, with the exception of 1070 reflections (5%) flagged for cross-validation purposes. No σ cutoff was applied. Successive rounds of manual rebuilding and refinement of the initial model resulted in *R* and *R*_free_ values of 0.2715 and 0.2786, respectively. The next ten cycles of simulated-annealing refinement in *phenix.refine* lowered the *R* factor to 0.2300. Implementation of TLS parameters, selected according to the *TLSMD* server (Painter & Merritt, 2005 ▶), and addition of H atoms at riding positions as a fixed contribution to *F*_c_ lowered the *R* factor below 0.2. Optimization of X-ray/stereochemistry weighting in *PHENIX*, refinement of the occupancies of some water molecules and several rounds of manual modelling resulted in final *R* and *R*_free_ values of 0.1694 and 0.2124, respectively. The final model consisted of residues 9–103 of chain *A*, residues 9–102 of chain *B* and 154 water molecules. The refinement statistics are given in Table 1 ▶. Structural illustrations were prepared with *PyMOL* (DeLano, 2002 ▶).

## 3. Results and discussion

### 3.1. Overall characteristics of the crystal structure

**Figure 1:**
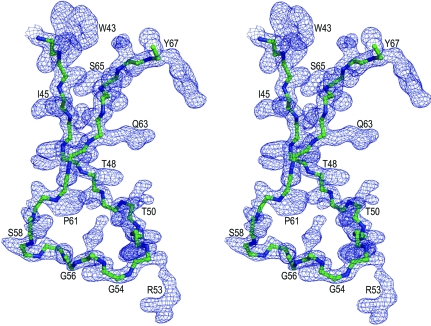
Stereoview of the main-chain trace of the flap loop plus flanking residues (Trp43–Tyr67). This trace of the flap of molecule *A* is shown in 2*F*_o_ − *F*_c_ electron density contoured at 1.0σ. Side-chain atoms have been omitted for clarity.

Despite its use during crystallization, the inhibitor is not present in the crystal structure and the protein exists in a monomeric fold. There are two independent 13PR molecules (*A* and *B*) in the asymmetric unit. They are virtually identical (C^α^ r.m.s.d. of 0.18 Å) and have the general chain topology known from the structures of dimeric retropepsins (Miller *et al.*, 1989 ▶; Wlodawer *et al.*, 1989 ▶). The polypeptide chains have excellent electron density for all structural elements, except for the N-terminus (residues 1–8) and C-terminus (104–114). The residues forming the flap loops show increased mobility (especially at the tips; Gln57–Ser58), which is visible as higher *B* factors, but there is no ambiguity about the tracing of these loops (Fig. 1 ▶) and their identical conformation in both molecules.

### 3.2. Conformation of the M-PMV PR monomer

The secondary structure assigned using *DSSP* (Kabsch & Sander, 1983 ▶) illustrates that the pseudo-twofold symmetry noted earlier in the protomers of retroviral proteases (Miller *et al.*, 1989 ▶) is preserved quite well in M-PMV PR. Notably, there is a helical segment present in the N-terminal half of the protein (Leu36–Asp38), a feature that replicates the canonical C-terminal helix (Arg95–Leu98) but which so far has only been found in EIAV (equine infectious anaemia virus) PR (Kervinen *et al.*, 1998 ▶). The C-terminal α-helix, however, is shorter than in most retropepsins.

### 3.3. The flap loop

**Figure 2:**
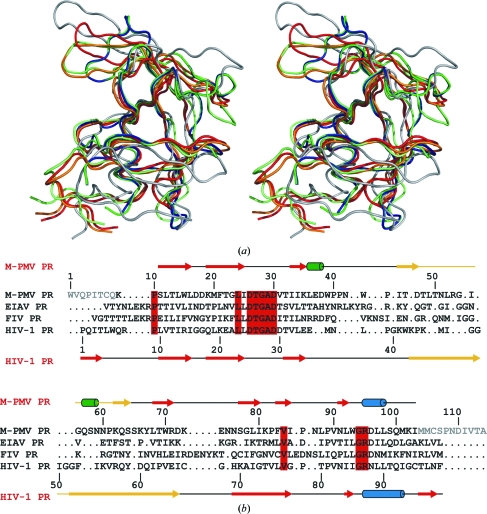
Alignment of retroviral proteases. (*a*) Stereoview of the superposition of the C^α^ traces of protomers of retroviral proteases: green and blue, M-PMV (*A* and *B*); red, HIV-1, apo form (PDB entry 3hvp); orange, HIV-1, inhibitor complex (PDB entry 4hvp); lime, EIAV (PDB entry 2fmb); grey, M-PMV, NMR model (PDB entry 1nso), energy-minimized (in water). (*b*) Structure-based sequence alignment of the M-PMV, EIAV (PDB entry 2fmb; lowest core C^α^ r.m.s.d.; Table 2 ▶), FIV (PDB entry 4fiv; highest level of sequence identity – 26.6%) and HIV-1 (PDB entry 3hvp) proteases. Residue numbers and secondary-structure elements (arrows, β-strands; blue, α-helices; green, 3_10_-helices; yellow, flap loops) are marked for the M-­PMV and HIV-1 proteases. Residues that are identical in all four sequences are shown on a red background. Disordered residues missing from the M-PMV PR structure are shown in grey.

The flap of M-PMV PR (residues Ile45–Ser64; Fig. 2 ▶ *b*) has a peculiar shape. It is not a smooth hairpin with β-type interactions as in other retropepsins, but has a wide conformation with a 3_10_-helical segment (Gln57–Asn59) present in its C-­terminal part. The flap folds upon the body of the protein but in a way that is different from the ‘lowered’ flap position over the active site of retropepsin dimers in complex with inhibitors (Fig. 2 ▶ *a*). The flap arm appears to be much shorter because of the helical insertion and its blunt end. The leading/trailing strands follow the ‘lowered’/‘open’ flap traces of HIV-1 PR. The 3_10_-helix in the trailing strand resembles a helical insertion in the flap of HTLV-1 (human T-cell leukaemia virus type-1) PR (Li *et al.*, 2005 ▶).

### 3.4. The active-site loop

**Figure 3:**
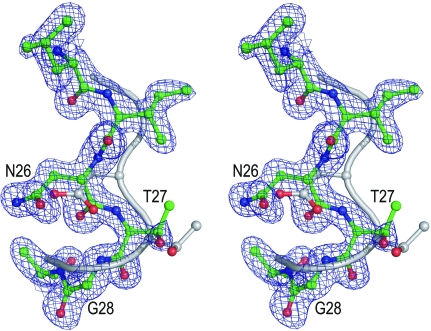
Stereoview of overlay of the active-site (D/N)TG loops of HIV-1 PR (PDB entry 3hvp, grey) and M-PMV PR (green) based on C^α^ superposition of the entire molecules. The M-PMV PR structure is shown as 2*F*_o_ − *F*_c_ electron density contoured at 1.3σ.

The active-site loop with the DTG (here NTG) triad has the general conformation as in other pepsins. However, in the absence of its replica, the key interactions (O^δ1^⋯Wat⋯O^δ1^, ‘fireman’s grip’) are missing and the side chains of Asn26 and Thr27 form only weak (∼3 Å) contacts with water molecules. On close com­parison, the loop deviates significantly from the trace in HIV-1 PR (Fig. 3 ▶); the C^α^ deviations culminate (2.1 Å) at Asn26, with the departure of the O^δ1^ atom being even larger (2.7 Å). This indicates that fine-tuning of the active-site geometry of retropepsins is only possible upon dimerization.

### 3.5. Comparison with other models of retropepsins

In C^α^ superpositions, the monomer of M-PMV PR shows marked departures from the sub­unit folds of dimeric retropepsin structures in the PDB (Table 2 ▶), with the most pro­nounced differences seen in the flap region. The core C^α^ atoms have r.m.s. deviations of ∼2 Å, but when all C^α^ atoms are included the deviations are much larger (≥3.5 Å), explaining the failure of the MR calculations. The ASV (avian sarcoma virus) PR model 2rsp (Jaskólski *et al.*, 1990 ▶) has an artificially low r.m.s.d. value (1.54 Å) because of its missing flaps. Of all the retropepsin protomers (as well as homologous proteins and domains; Table 2 ▶), the closest structural homologue is the protein from HTLV-1, but the best agreement in the core region is with EIAV PR. The *Foldit* model used to solve the structure by MR (Khatib *et al.*, 2011 ▶) has a similar core r.m.s.d. as the crystallographic models of retropepsins but the value calculated for all C^α^ atoms is significantly improved, reflecting *inter alia* that the flap has a generally correct conformation.

On the background of the numerous superpositions with crystallographic models of retropepsins (Fig. 2 ▶ *a*), the similarity to the NMR model of M-PMV 12PR in the core region is the lowest. Here again the flap shows a widely different conformation, but even with its exclusion the match of the protein core is inferior. The alignments reported in Table 2 ▶ were calculated for two energy-minimized models of the NMR coordinates 1nso kindly provided by Dr Richard Hrabal. These results explain why the NMR structure 1nso failed to solve the crystal structure directly as an MR model. Incidentally, a similar r.m.s.d. value is obtained for the only other NMR structure of a retroviral protease (from simian foamy virus) monomer in the PDB (PDB entry 2jys; Hartl *et al.*, 2008 ▶).

### 3.6. Structural consequences of the monomeric fold

There is no question about the absence of proper biologically competent dimers of the protease in this crystal structure because the N- and C-terminal peptides, which are absolutely required for and highly ordered upon dimerization, are totally disordered. The disordered fragments include the cysteine residues Cys7 and Cys106 (here mutated to Ala) which are known to connect the termini under non­reducing conditions. The existence of the Cys7–Cys106 bond has been demonstrated in monomeric M-PMV PR, but it can be envisaged that it could be reconfigured into an intermolecular context upon dimerization, as the canonical topology of the dimeric interface is N(*A*)–C(*B*)–C(*A*)–N(*B*). The novel type of interface reported recently for XMRV (xenotropic murine leukaemia virus-related virus) PR (Li *et al.*, 2011 ▶) is not applicable in this case as it does not include the N-terminal peptide at all. In the intramolecular context, the Cys7–Cys106 disulfide stabilizes the monomeric fold, while in the intermolecular context it would be expected to reinforce the dimer. Indeed, it has been shown that in the C7A/C106A mutant the enzymatic activity is reduced *in vitro* by 60% (Zábranská *et al.*, 2007 ▶). However, *in vivo* these mutations do not influence Gag processing and virus infectivity. Since the reversible oxidation of immature M-­PMV particles has been shown to regulate PR activation *in vitro* (Parker & Hunter, 2001 ▶), one can speculate that other cysteines in the Gag polyproteins also participate in PR activation by modulating the conformation and accessibility of the PR cleavage sites or by regulating the binding of cellular proteins that could protect the polyproteins from premature processing.

**Figure 4:**
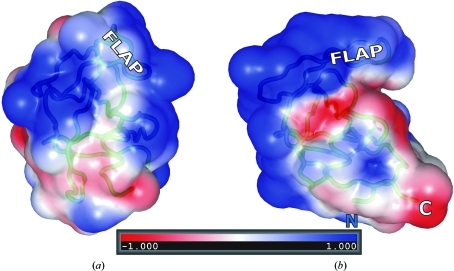
Electrostatic potential surface of retroviral protease protomers. The M-PMV PR monomer (*a*) is shown in the same orientation and on the same scale as the HIV-1 PR protomer (*b*) extracted from the dimeric molecule (PDB entry 3hvp). The complete HIV-1 PR dimer is generated by the action of a vertical dyad, which creates a second copy facing the first molecule on the right. In this view, the N- and C-­termini (missing in M-PMV PR) are at the bottom and the flap loops are at the top. The active-site cavity is marked by the Asn26/Asp25 residue (ball-and-stick representation). In M-PMV PR the cavity is completely covered by the curled flap. The area of positive potential on this M-PMV PR surface is influenced by the D26N substitution, but it is of note that this mutation does not influence the tendency of the protein to fold as a monomer. The electrostatic potential (negative, red; positive, blue) was calculated in *APBS* (Baker *et al.*, 2001 ▶).

When the present M-PMV PR molecule is viewed from the direction of its absent dimerization partner, one sees a uniformly positively charged surface (Fig. 4 ▶). This is different from a similar view of the HIV-1 PR protomer, in which both charges and hydrophobic patches are seen, and may partly explain why in the absence of substrate/inhibitor the M-PMV protein can stably exist as a monomer, at least with the D26N mutation. Fig. 4 ▶ also illustrates that the curled flap closely covers the active-site cavity, while in the HIV-1 PR protomer extracted from the dimeric enzyme the cavity would be freely accessible.

### 3.7. Crystal packing and molecular interactions

**Figure 5:**
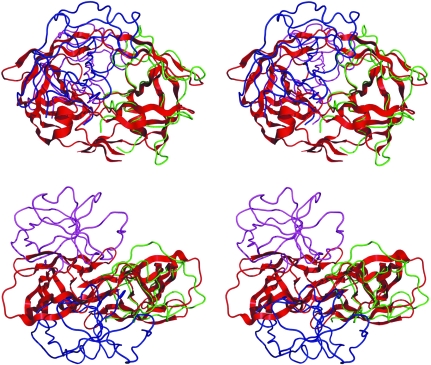
Stereoview of superposition of the C^α^ atoms of the HIV-1 PR protomer (PDB entry 3hvp, red) on monomer *A* of M-PMV PR in the crystal structure (green). This superposition illustrates the relation of the HIV-1 PR dimer (cartoon) to the neighbouring copies of M-PMV PR monomer *B* in the crystal (blue and magenta). Bottom panel, view down the twofold axis of the HIV-1 PR dimer; top panel, a perpendicular view with the twofold axis vertical.

The crystal packing is very dense, with only 28.1% of the unit-cell volume occupied by solvent (Table 1 ▶). Despite this, the two protein molecules in the asymmetric unit do not form a tight intimate dimer (see above). However, the polypeptide chains *A* and *B* do form crystal contacts (Fig. 5 ▶) that, according to *PISA* (Krissinel & Henrick, 2007 ▶), ‘are not strongly indicative of complex formation in solution’. These contacts bury <800 Å^2^ of surface area per monomer (for reference, HIV-1 PR dimerization buries ∼1700 Å^2^ per monomer) and are formed in a mutual fashion by interactions of the flap loop with loop-80 (Pro86–Val90). Loop-80 is an important element of the retropepsin structure as it participates in shaping the inhibitor-binding cavity. Another discernible mode of crystal packing, involving an *a*-translated molecule *B*, buries ∼400 Å^2^ in contacts that are formed nearly exclusively by the flap loops. This is an intriguing observation because in dimeric retropepsins the flaps also contribute to protomer interactions (in addition to the N- and C-termini and the active-site loops), especially in complexes, when they are lowered onto the bound inhibitor. In general, the lattice contacts in the present structure tend to shield from solvent the face of the molecule that is normally buried upon dimerization. When a dimer of HIV-1 PR (PDB entry 3hvp; Wlodawer *et al.*, 1989 ▶) is superposed on molecule *A* of the present structure, it is obvious that the crystal-lattice aggregates of M-PMV PR are different from the functional retropepsin dimer. In particular, the active-site loops, which in the homodimer are closely associated through a ‘fireman’s grip’ and a water-mediated (or hydroxyl-mediated) contact between the catalytic aspartates, are far apart, with the C^α^⋯C^α^ distance between the Asn26 residues being 11.4 Å. It is evident from Fig. 5 ▶ that the monomers forming the crystallo­graphic aggregates of M-PMV PR remain associated by the flaps but are ‘pulled apart’ in the active site and N-/C-terminal areas. In other words, the protomers are in close proximity and quite well juxtaposed for productive association but still do not interdigitate their N-/C-termini in a proper dimeric association. One might speculate that the dimer does not assemble by side-by-side alignment of pre-formed monomeric proteins but is more likely to arise during the folding process that involves the formation of the dimer interface (from the N- and C-termini) at an early rather than late stage, as observed for HIV-1 PR (Ishima *et al.*, 2001 ▶).

## 4. Conclusions and outlook

The present structure shows that retroviral protease can fold and exist stably as a monomer. This lends support to the notion of using dimerization inhibitors as potential antiretroviral drugs. The disruption of the dimeric interface might, for instance, be achieved by complexing the protein with an oligopeptide with the N- and C-terminal sequences. In the case of M-PMV PR this should be even easier because one might exploit the potential to form a protease–inhibitor S—S bond, but a similar strategy would also be possible for HIV-1 PR, which contains a Cys95 residue at the C-terminus.

## Supplementary Material

PDB reference: monomeric M-PMV retroviral protease, 3sqf

## Figures and Tables

**Table 1:** Data-collection and structure-refinement statistics Values in parentheses are for the highest resolution shell.

Data collection	
Crystal dimensions (mm)	0.30 × 0.15 × 0.15
Space group	*P*2_1_
Unit-cell parameters (Å, °)	*a* = 26.76, *b* = 86.62, *c* = 39.31, β = 104.6
Solvent content (%)	28.1
Temperature (K)	100
X-ray source	EMBL/DESY X13
Wavelength (Å)	0.8086
Oscillation angle (°)	0.5
No. of frames	456
Resolution (Å)	43.3–1.63 (1.73–1.63)
Mosaicity (°)	0.28
*R*_int_†	0.068 (0.752)
*R*_meas_‡	0.076 (0.860)
〈*I*/σ(*I*)〉	14.9 (1.9)
Reflections	
Measured	99683
Unique	21369
Completeness (%)	99.0 (96.3)
Multiplicity	4.7 (4.2)
Wilson *B* factor (Å^2^)	26.0
Refinement	
Resolution (Å)	28.6–1.63
No. of reflections	
Work set	20295
Test set	1070
*R*/*R*_free_§	0.1694/0.2124
Protein molecules in asymmetric unit	2
No. of atoms	
Protein	1527
Water	154
〈*B* factor〉 (Å^2^)	
Protein	28.4
Water	34.6
R.m.s. deviations from ideal	
Bond lengths (Å)	0.018
Bond angles (°)	1.77
Ramachandran statistics (%)	
Favoured	96.8
Allowed	3.2
Outliers	0.0
PDB code	3sqf

† *R*_int_ = 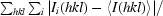 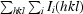, where *I_i_*(*hkl*) is the *i*th measurement of the intensity of reflection *hkl* and 〈*I*(*hkl*)〉 is the mean intensity of reflection *hkl*.

‡ *R*_meas_ = 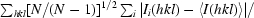 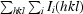, where *I_i_*(*hkl*) is the *i*th measurement of the intensity of reflection *hkl*, 〈*I*(*hkl*)〉 is the mean intensity of reflection *hkl* and *N* is the number of observations of intensity *I*(*hkl*) (multiplicity).

§ *R* = 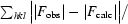 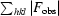, where *F*_obs_ and *F*_calc_ are the observed and calculated structure factors, respectively. *R*_free_ was calculated analogously for a randomly selected 5% of the reflections.

**Table 2:** R.m.s.d. values (Å) for core C^α^ superpositions of molecule *A* of M-PMV PR on molecule *B* and on protomers of aspartic retroviral proteases, N- and C-terminal domains of porcine pepsin and the retropepsin-like putative protease domain of the eukaryotic protein Ddi1 (PDB codes are given in parentheses) R.m.s.d. values for core C^α^ atoms are shown in the first row and were calculated using the *SSM* server (Krissinel & Henrick, 2004 ▶). Values in the second row are for all common C^α^ atoms (calculated in *ALIGN*; Cohen, 1997 ▶). The coordinates of the NMR model 1nso were energy-minimized *in vacuo* and in water. The following abbreviations are used to identify different retroviral proteases: M-PMV, Mason–Pfizer monkey virus; HIV-1, human immunodeficiency virus type 1; SIV, simian immunodeficiency virus; ASV, avian sarcoma virus; FIV, feline immunodeficiency virus; EIAV, equine infectious anaemia virus; HTLV-1, human T-cell leukaemia virus type 1; XMRV, xenotropic murine leukaemia virus-related virus.

M-PMV														
		NMR (1nso)											Pepsin (4pep)	
*B*	*Foldit*	Vacuo	Water	HIV-1 (3hvp)†	HIV-1 (4hvp)‡	SIV (1yth)‡	ASV (2rsp)†§	FIV (4fiv)‡	EIAV (2fmb)‡	HTLV-1 (3liy)‡	XMRV (3nr6)†	Ddi1 (2i1a)	N	C
0.18	2.08	3.04	2.57	2.14	2.17	2.09	1.54	1.94	1.65	2.05	1.95	2.23	4.17	3.13
0.18	2.87	5.51	4.33	8.92	9.06	7.73	7.95	4.28	8.23	3.50	10.49	9.77	4.44	5.93

† Apo form.

‡ Inhibitor complex.

§ Flap loops missing.
